# Interspecies Comparison of Interaction Energies between Photosynthetic Protein RuBisCO and 2CABP Ligand

**DOI:** 10.3390/ijms231911347

**Published:** 2022-09-26

**Authors:** Masayasu Fujii, Shigenori Tanaka

**Affiliations:** Department of Computational Science, Graduate School of System Informatics, Kobe University, 1-1 Rokkodai, Nada-ku, Kobe 657-8501, Japan

**Keywords:** ribulose-1,5-bisphosphate carboxylase/oxygenase (RuBisCO), fragment molecular orbital (FMO) method, inter-fragment interaction energy (IFIE), singular value decomposition (SVD)

## Abstract

Ribulose 1,5-bisphosphate carboxylase/oxygenase (RuBisCO) functions as the initial enzyme in the dark reactions of photosynthesis, catalyzing reactions that extract CO_2_ from the atmosphere and fix CO_2_ into organic compounds. RuBisCO is classified into four types (isoforms I–IV) according to sequence-based phylogenetic trees. Given its size, the computational cost of accurate quantum-chemical calculations for functional analysis of RuBisCO is high; however, recent advances in hardware performance and the use of the fragment molecular orbital (FMO) method have enabled the ab initio analyses of RuBisCO. Here, we performed FMO calculations on multiple structural datasets for various complexes with the 2′-carboxylarabinitol 1,5-bisphosphate (2CABP) ligand as a substrate analog and investigated whether phylogenetic relationships based on sequence information are physicochemically relevant as well as whether novel information unobtainable from sequence information can be revealed. We extracted features similar to the phylogenetic relationships found in sequence analysis, and in terms of singular value decomposition, we identified residues that strongly interacted with the ligand and the characteristics of the isoforms for each principal component. These results identified a strong correlation between phylogenetic relationships obtained by sequence analysis and residue interaction energies with the ligand. Notably, some important residues were located far from the ligand, making comparisons among species using only residues proximal to the ligand insufficient.

## 1. Introduction

**Figure 1:**
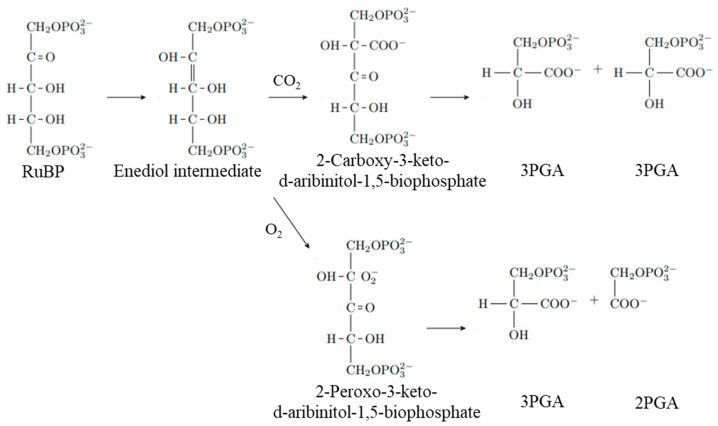
The carboxylation and oxygenation reactions of RuBisCO. RuBP reacts with CO_2_ to form two molecules of 3PGA. Alternatively, RuBP reacts with O_2_ to form one molecule of 3PGA and a molecule of 2PGA. RuBP, d-ribulose 1,5-bisphosphate; 3PGA, 3-phospho-d-glycerate; 2PGA, 2-phosphoglycolate.

Ribulose 1,5-bisphosphate (RuBP) carboxylase/oxygenase (RuBisCO) is involved in the fixation of CO_2_ in the Calvin cycle [1]. RuBisCO is a seemingly inefficient enzyme despite the fact that it appeared >3 billion years ago and has been affected by natural selection. There are two reasons for the relatively low reaction efficiency. The first is that the reaction is not fast, with a turnover rate of ~4 s^−1^ for carboxylation, whereas most enzymatic reactions have turnover rates ranging from 10^4^ to 10^5^ s^−1^ [2]. Second, RuBisCO not only catalyzes the reaction between RuBP and CO_2_ (carboxylase reaction) but also the reaction between RuBP and O_2_ (oxygenase reaction). Because these reactions are competitively catalyzed at the same site in RuBisCO, the carboxylase reaction is inhibited by the oxygenase reaction (Figure 1). Several studies report that the low reaction efficiency of RuBisCO may be due to a trade-off between its reaction rate and ability to discriminate between CO_2_ and O_2_ (substrate specificity) [2,3]. The reaction rate and substrate specificity of RuBisCO somewhat vary among photosynthetic organisms, and interspecies differences have also been studied [4,5,6].

**Figure 2:**
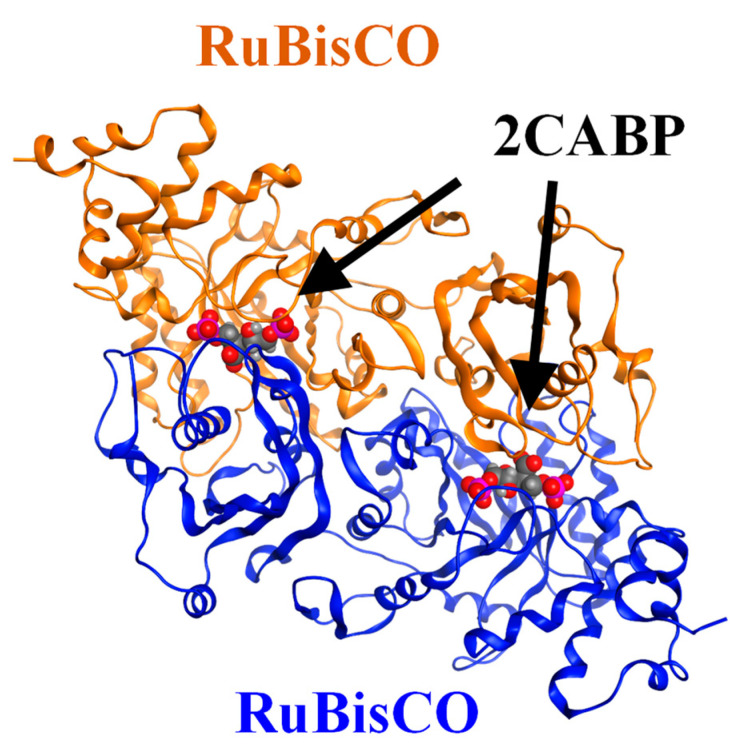
Crystal structure of RuBisCO complexed with a 2CABP ligand (Form II, PDB-ID: 4LF1). Ribbons (blue and brown) represent the L_2_ dimer, and red spheres represent 2CABP. 2CABP, 2′-carboxylarabinitol 1,5-bisphosphate; PDB, Protein Data Bank.

RuBisCO catalysis occurs at the interface of two large (L) subunits (LSU; ~50–52 kDa) (Figure 2). The L_2_ dimer is supposed to be a functional unit that contains two active sites. The phylogenetic relationship of RuBisCO was classified into forms I through IV on a sequence-based phylogenetic tree, with the species belonging to each form sharing common features. Form I RuBisCOs include higher plants, cyanobacteria, and algae, in which a tetramer core of L_2_ dimers is capped at both poles by four small (S) subunits (SSU; 15kDa), thus resulting in the L_8_S_8_ structure. Form II RuBisCOs include bacteria, and form III RuBisCOs include (non-photosynthetic) archaea, which exist as L_2_ dimers or oligomers of L_2_ dimers. Form III RuBisCOs do not contribute to the photosynthetic reaction process but can functionally substitute for photosynthetic RuBisCOs. For example, *Thermococcus kodakarensis*, which possesses a form III RuBisCO, is suggested to function in a metabolic pathway involved in the degradation of nucleoside 5′-phosphate [7,8]. Form IV RuBisCOs are RuBisCO homologues or RuBisCO-like proteins that lack some residues essential for the RuBisCO reaction. For example, the RuBisCO homologues present in *Bacillus subtilis* are reportedly active in the methionine-salvage pathway [9]. Furthermore, some species for which it is difficult to express phylogenetic relationships using conventional sequence-based classification on RuBisCO have been discovered [10,11,12].

Understanding the evolutionary history of RuBisCO provides clues to the constraints of the RuBisCO reaction. By creating a dendrogram using features different from the amino acid sequences, we hypothesized that we could augment conventional sequence-based classification of RuBisCOs and potentially discover features that could not be extracted by sequences alone, which might provide new insights into RuBisCO evolution.

Recent improvements in hardware performance and the use of the fragment molecular orbital (FMO) method [13,14], which performs quantum-chemical calculations in a highly parallelized manner, have enabled ab initio calculations for the L_2_ dimer, a common functional unit of RuBisCO and RuBisCO-like proteins, in relatively short time. To investigate the phylogenetic relationships of various RuBisCO species, we focused on the inter-fragment interaction energy (IFIE) between the 2′-carboxylarabinitol 1,5-bisphosphate (2CABP) ligand and each amino acid residue of the RuBisCO protein obtained by FMO calculations, where we hypothesized that we could infer the essential information from the analysis on L_2_ dimer. We then clarified whether the IFIEs were related to the phylogenetic relationships in the sequences or whether there was any information that could not be obtained exclusively from sequence data.

Thus, the purpose of this study is to elucidate the relationship between the substrate-residue interactions and the enzymatic evolution of RuBisCO. Employing the combination of the FMO-IFIE scheme and the multivariate analysis, we present the details of our results with the FMO calculations and discuss the possibility of classifying RuBisCO isoforms in terms of residue–ligand interactions.

## 2. Results and Discussion

### 2.1. Remark on FMO Calculation Results for the RuBisCO–2CABP System

**Figure 3:**
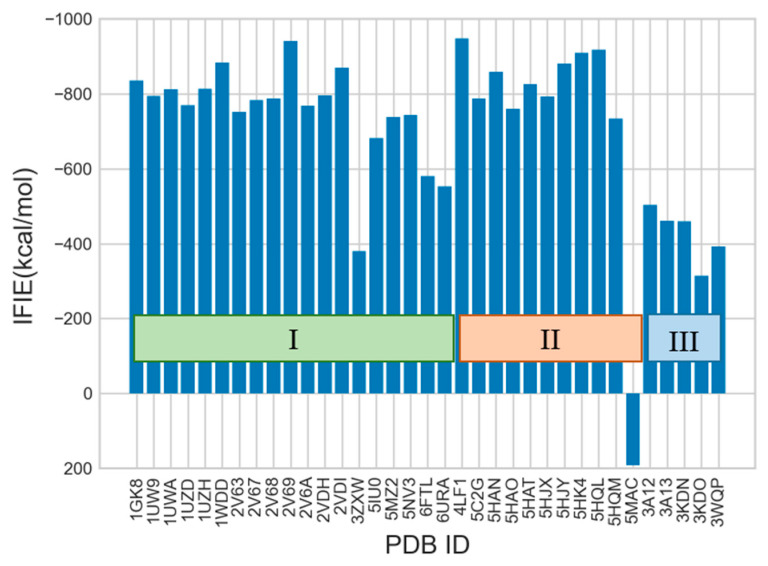
Calculated results of IFIE-sum for 2CABP. The PDB structures of 35 RuBisCOs are grouped into three isoforms I–III.

The FMO results of IFIE-sum for the interactions between 2CABP ligand and 1115 residues of L_2_ dimer of 35 RuBisCOs is illustrated in Figure 3. In general, the absolute values of the IFIE-sum tended to be smaller in form III than in forms I and II. The present examination of the IFIE-sum showed that *Methanococcoides burtonii* RuBisCo (PDB entry: 5MAC; referred to as 5MAC hereafter) exceptionally had a repulsive interaction with the ligand, unlike other structures.

According to a previous study [10], 5MAC was classified as form II in the sequence-based phylogenetic tree, but its function was closer to that of form III because 5MAC functions in purine/pyrimidine metabolism, which is different from the photosynthetic reaction. However, 5MAC has a unique 29-amino acid sequence insertion, which serves to connect the L_2_ dimer with another L_2_ dimer and Mg^2+^, which is present separately from the active sites.

Therefore, for more dependable FMO analysis, it would be necessary to include not only the L_2_ dimer but also the entire oligomerized RuBisCO and Mg^2+^ that exists between the dimers in the case of 5MAC. In fact, calculation using only the L_2_ dimer showed that the IFIE-sum with 58 residues characteristic of 5MAC (2 × 29 residues because of the dimer) was 304 kcal/mol, which was strongly repulsive to the ligand. Because the calculation conditions were aligned with those of the L_2_ dimer in this study, we considered that 5MAC could not be accurately analyzed. For these reasons, we excluded 5MAC from subsequent analyses.

### 2.2. Comparison of the Sequence-Based Phylogenetic Tree and IFIE-Based Dendrogram

**Figure 4:**
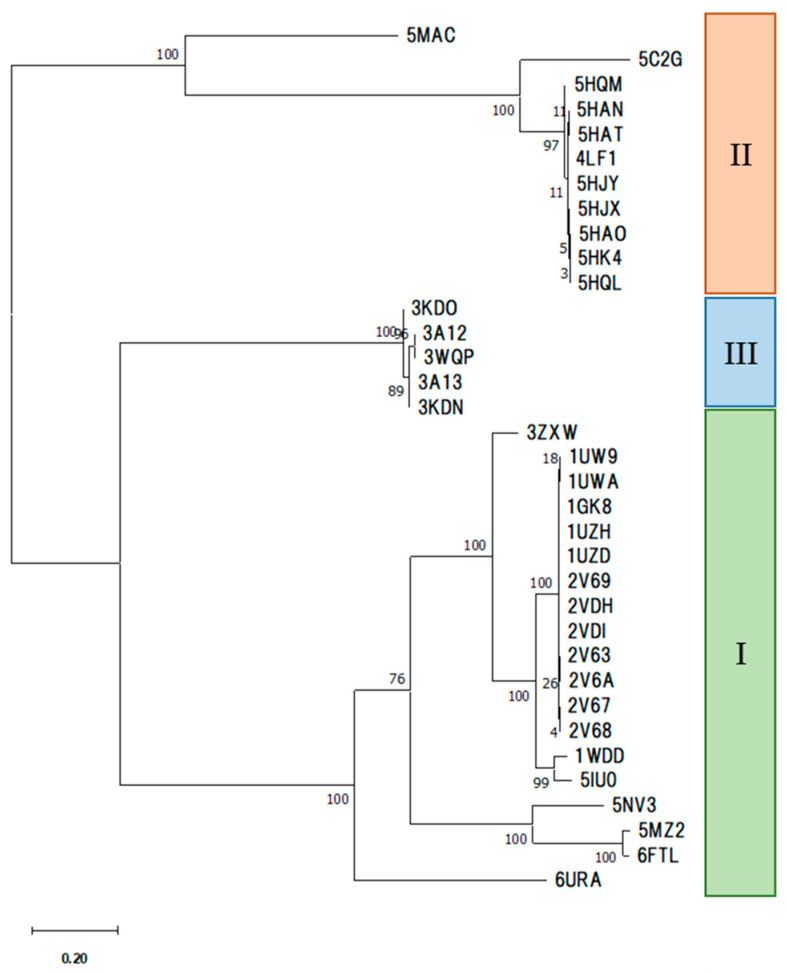
Phylogenetic tree of RuBisCOs obtained through multiple alignment of amino acid sequences. The corresponding PDB entries are shown along with their isoform (I–III) specification.

First, in terms of multiple alignments of amino acid sequences, we constructed a phylogenetic tree that focused on forms I through III (Figure 4). The sequence length of the alignment was 556 sites (for LSU monomer), of which 171, 154, and 432 sites were conserved in forms I, II, and III, respectively, with 59 sites conserved in all species.

**Figure 5:**
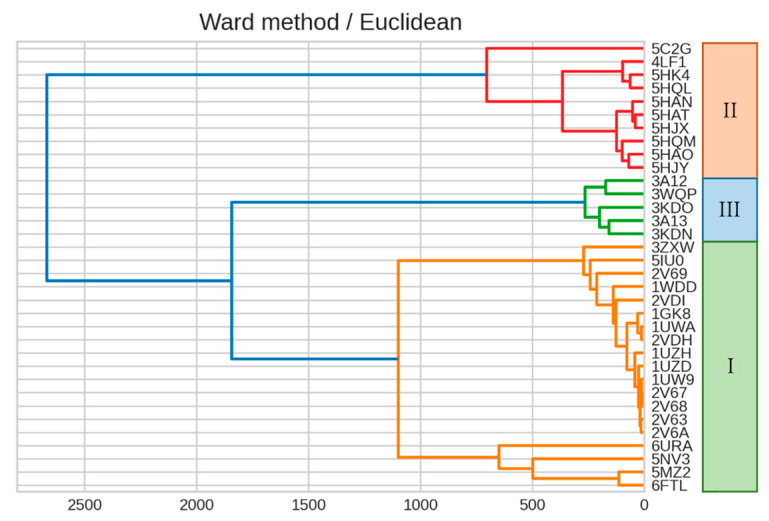
Dendrogram generated from the clustering result on IFIEs using Ward’s method. Color coding is set at 60% of the maximum Euclidean distance. The corresponding isoform specifications, I–III, identified by the sequence alignment (see Figure 4) are also shown.

We then constructed a dendrogram by clustering a RuBisCO IFIE matrix using Ward’s method (Figure 5). The classification of isoforms and phylogenetic relationships within the forms revealed that the dendrogram generated by the IFIEs was similar to the sequence-based phylogenetic tree shown in Figure 4.

### 2.3. Singular Value Decomposition (SVD) Analysis

**Figure 6:**
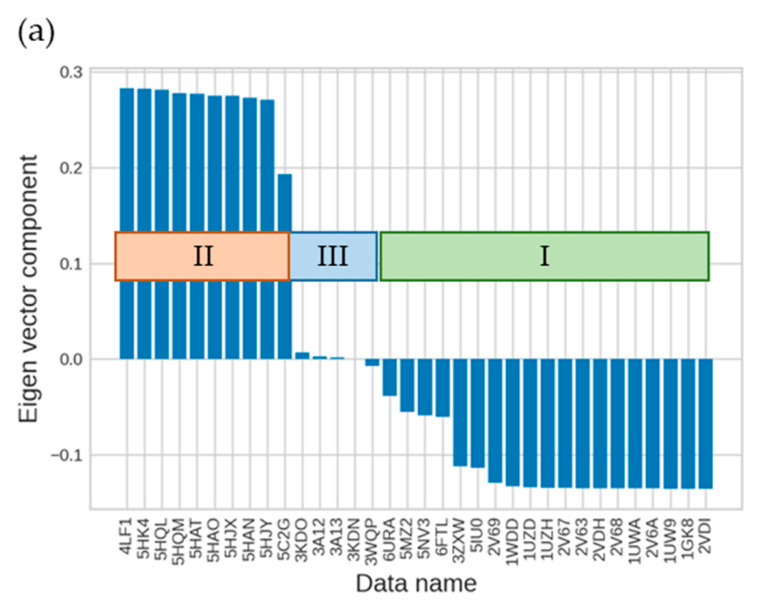

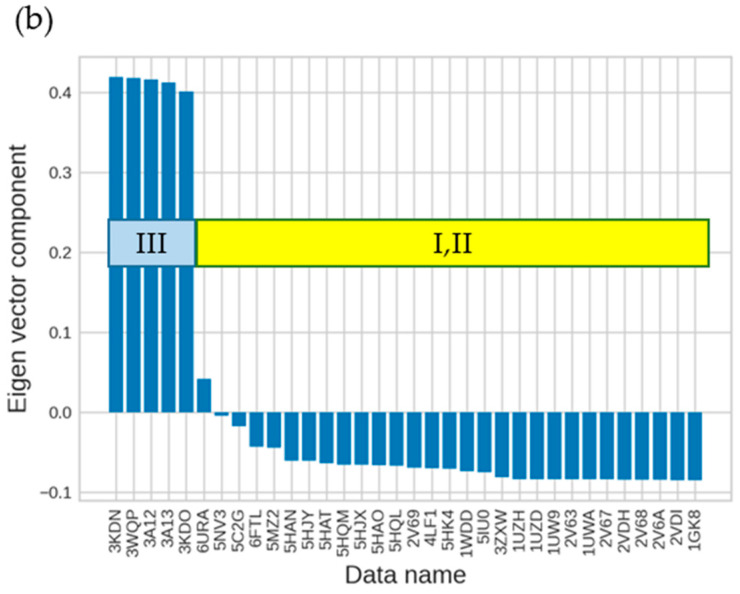
Results of SVD analysis for the right-singular vectors of IFIE matrix. (**a**) The second and (**b**) third right-singular vectors for each structure dataset identified by PDB entry. The corresponding isoforms I–III are also indicated.

We then applied the singular value decomposition (SVD) technique (see Section 3.3) to the RuBisCO IFIE matrix to obtain more insights. By checking the right-singular vectors, the second and third singular vectors represent the difference in forms and the difference between forms III and I and II, respectively (Figure 6). Additionally, by checking the left-singular vector, we found that the first singular vector represented the average value of the IFIEs (see Table 1 and Table 2, and details are shown in Figures S1 and S2 in Supplementary Materials), which were predominantly governed by electrostatic interactions. These results were similar to those of previous studies that used other proteins [15].

Evaluation for the eight residue sites with large absolute eigenvector values that constituted the second singular vector (Table 1) identified seven sites that were mostly conserved within each form but not in all RuBisCO data (Table 3). Here, we could not consider the site number 582 as an important site of the second singular vector because there was a gap in PDB structures corresponding to the sequences in forms II and III. Although the residues at site number 332 were conserved in many species regardless of form, structural investigations showed a difference in histidine (electrically neutral in forms I and III but showing a charge of +1 in form II). The differences among the forms in the eight sites with the largest absolute values that constituted the second singular vector were primarily due to differences in the electric charge of the residues. Therefore, we identified the sites with large absolute values in the second singular vector as characteristic sites in the forms, which indicated that the common features in the phylogenetic tree based on the sequences were important.

**Figure 7:**
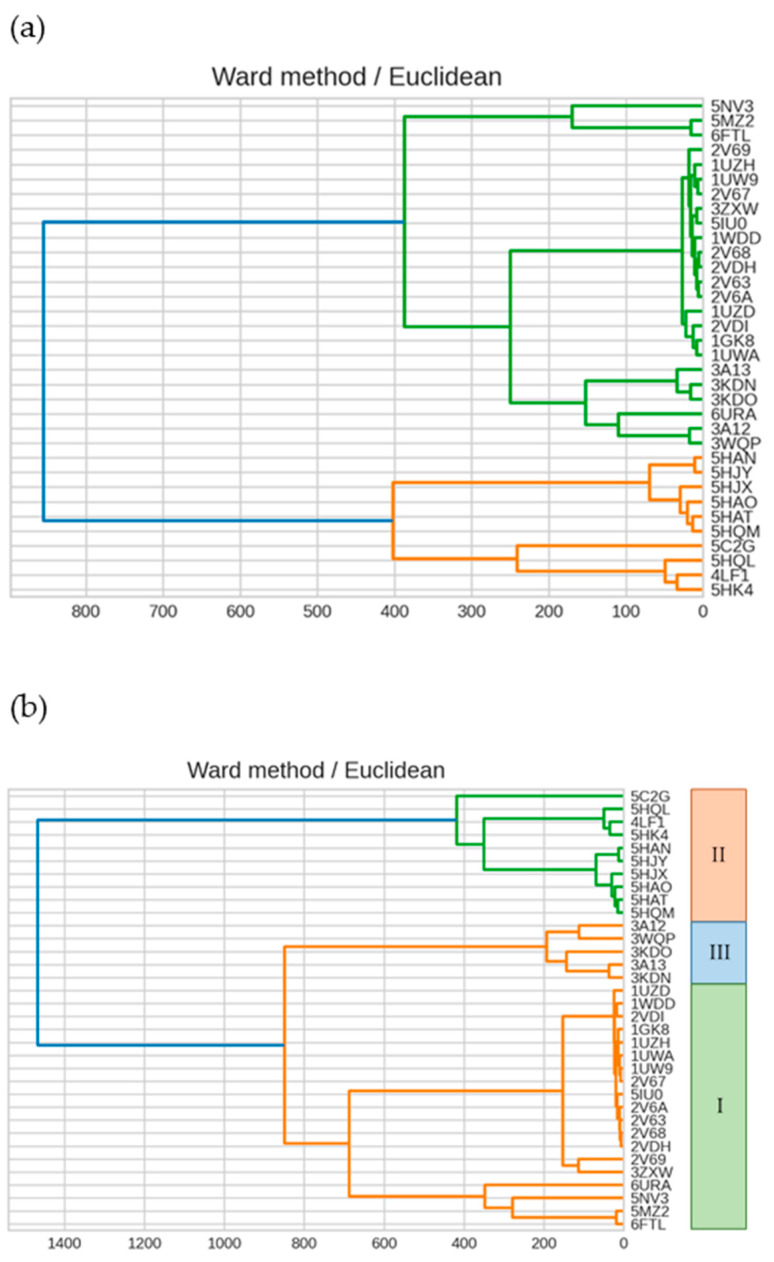
Dendrogram showing the clustering result on IFIEs using Ward’s method. Color coding is set at 60% of the maximum Euclidean distance. Dendrograms were created using (**a**) the top 20 sites and (**b**) the top 100 sites for the second left-singular vector.

Assessment of up to 20 sites with larger absolute values revealed the same trend. To determine the number of sites required to reproduce the sequence-based isoform classification, we increased the number of sites with large absolute values that made up the second singular vector to 20, 40, and 60 and then reviewed the respective dendrograms. We found that the shape of the tree was unstable unless ~100 sites were used and that it was impossible to reproduce the shape classification by sequences (Figure 7). Therefore, we concluded that a small number of sites would be insufficient to express differences in the forms.

**Figure 8:**
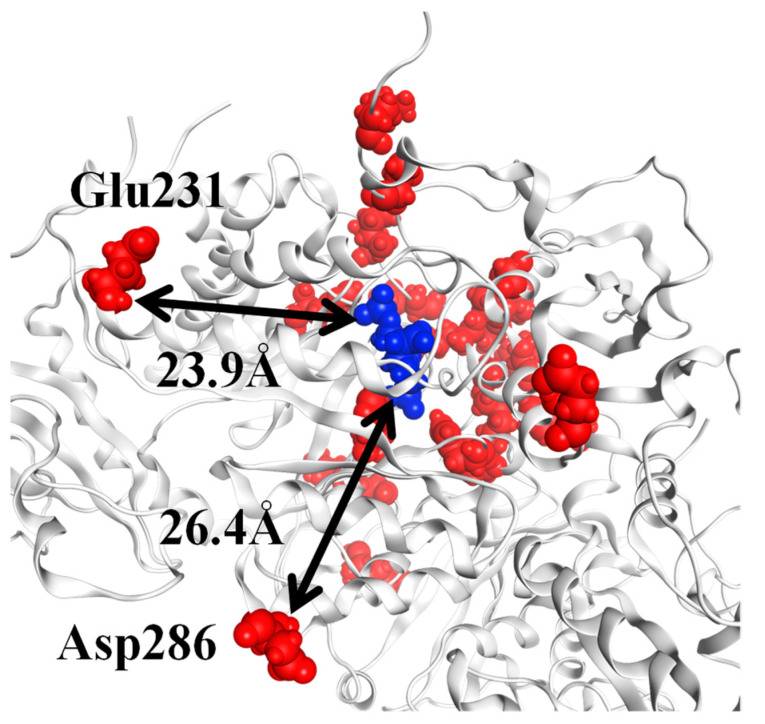
Location of the top 20 residues in the second left-singular vector. For the L_2_ dimer of RuBisCO (PDB ID: 1UWA, FMODB ID: 53J5Z), the 2CABP ligand is shown by blue spheres, and red spheres represent the residues corresponding to the top 20 sites for the second left-singular vector (A-chain: Glu231, Asp286, His298, Asp302, Arg303, His327, Lys356, Arg360, His386, Ile465, and Glu468; O-chain: Lys14, Gln45, Ala56, Thr68, Glu110, Lys128, Ala129, Arg131, and His307). The distance between 2CABP and Glu231 is 23.9 Å, and that between 2CABP and Asp286 is 26.4 Å.

Meanwhile, the positions of the 20 residues with the largest absolute values in the second singular vector were found to be significantly far from the ligand, with some located >20 Å away from the ligand (Figure 8). We thus concluded that the residues around the ligand alone were insufficient for interspecific comparison.

### 2.4. Dendrogram Generated by Residues Surrounding the Active Site

**Figure 9:**
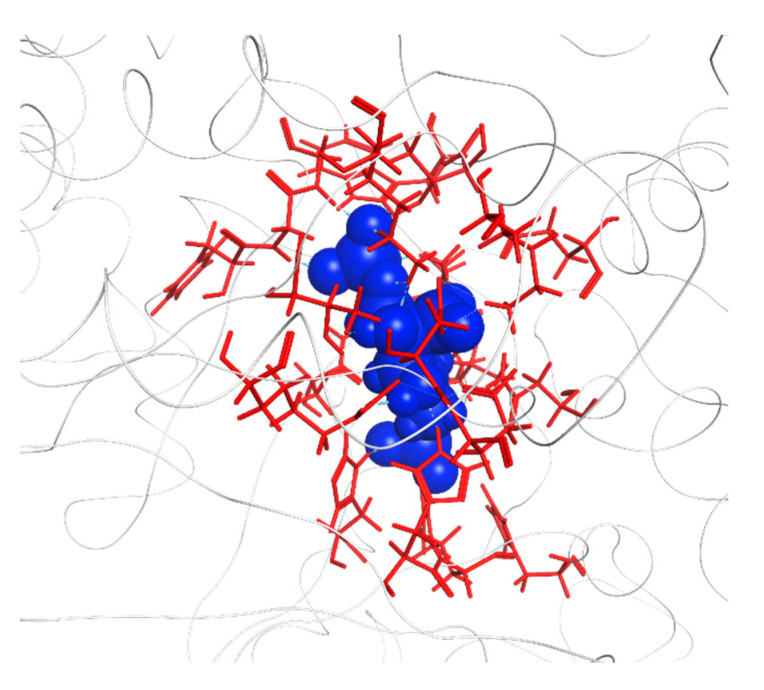
Residues around the active site (PDB entry: 1UWA, FMODB entry: 53J5Z), where the 2CABP ligand is shown by blue spheres. Red sticks represent 22 residues used for clustering (A-chain: Thr173, Lys175, Lys177, KCX201, Asp203, Glu204, His294, Arg295, His298, His327, Lys334, Leu335, Ser379, Gly380, Gly381, Phe402, Gly403, and Gly404; O-chain: Glu60, Thr65, Trp66, and Asn123). KCX, a lysine residue modified by carbamylation.

**Figure 10:**
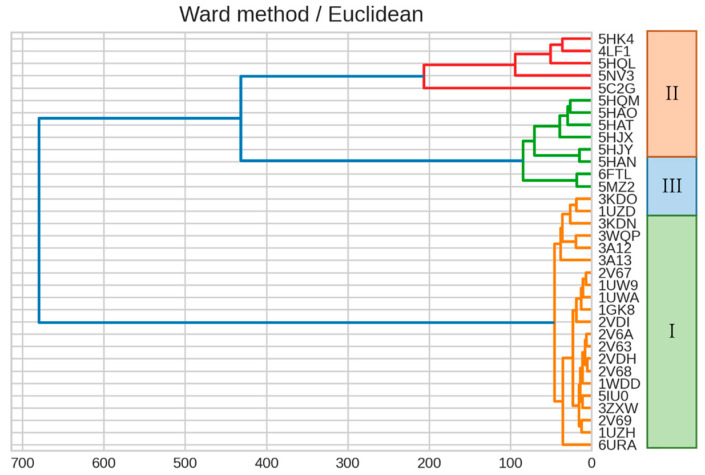
Dendrogram showing the clustering result on IFIE data using Ward’s method. Color coding is set at 60% of the maximum Euclidean distance. The dendrogram was created using 22 sites corresponding to residues surrounding the active site (see Figure 9).

Based on a previous study [16], we constructed a dendrogram by using sites corresponding to 22 residues surrounding the active site (see Figure 9 and Figure 10). While 16 sites were conserved in the form, these sites alone were insufficient for form classification. This result reinforced our conclusion that the residues around the active site alone are insufficient to explain the differences between the forms. The IFIE result thus provides a quantitative justification for the intuition that selection pressure is strong on amino acids of the active site essential for enzyme function, and possible mutations in evolution take place at those residue sites somewhat away from the active site. The present analysis then gives information on how distant and what concrete residues make major contributions to proper classification.

### 2.5. Comparison of Features with Different Preprocessing Methods

We then investigated whether alternative preprocessing of IFIEs resulted in differences in the extraction of features using phylogenetic relationships with SVD. We applied the following normalization procedure for preprocessing:

(1) $$IFIE^{\prime }=\frac{IFIE-IFIE_{avg}}{IFIE_{std}}$$

where ${IFIE}^{\prime }$ is the normalized IFIE, IFIE is the original IFIE, $IFIE_{avg}$ is the average IFIE per site, and $IFIE_{std}$ is the standard deviation per site.

**Figure 11:**
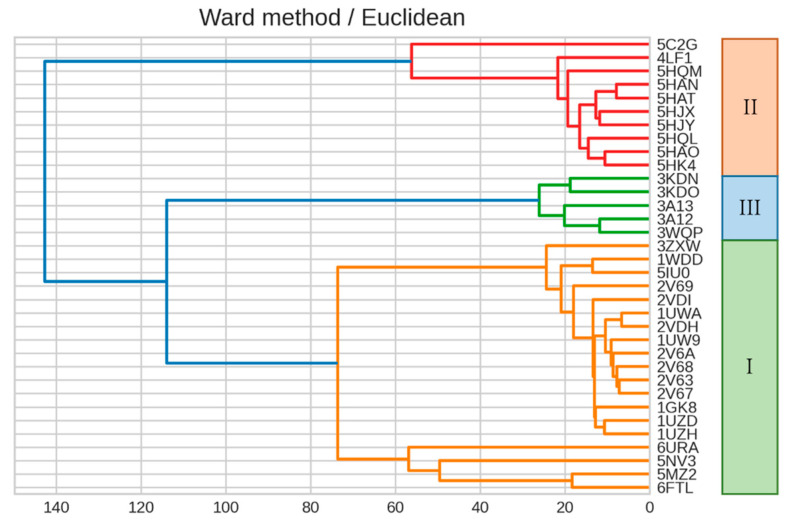
Dendrogram showing the clustering result using Ward’s method, where the dendrogram was created using normalized IFIE data. Color coding is set at 60% of the maximum Euclidean distance.

Normalization produces a dendrogram in which the effect of sites with large absolute values of IFIE is mitigated. Before normalization, all sites with all IFIEs < 1 kcal/mol were excluded as the sites with considerable noise, resulting in 298 excluded sites. Clustering revealed a dendrogram similar to that obtained without normalization (Figure 11).

**Figure 12:**
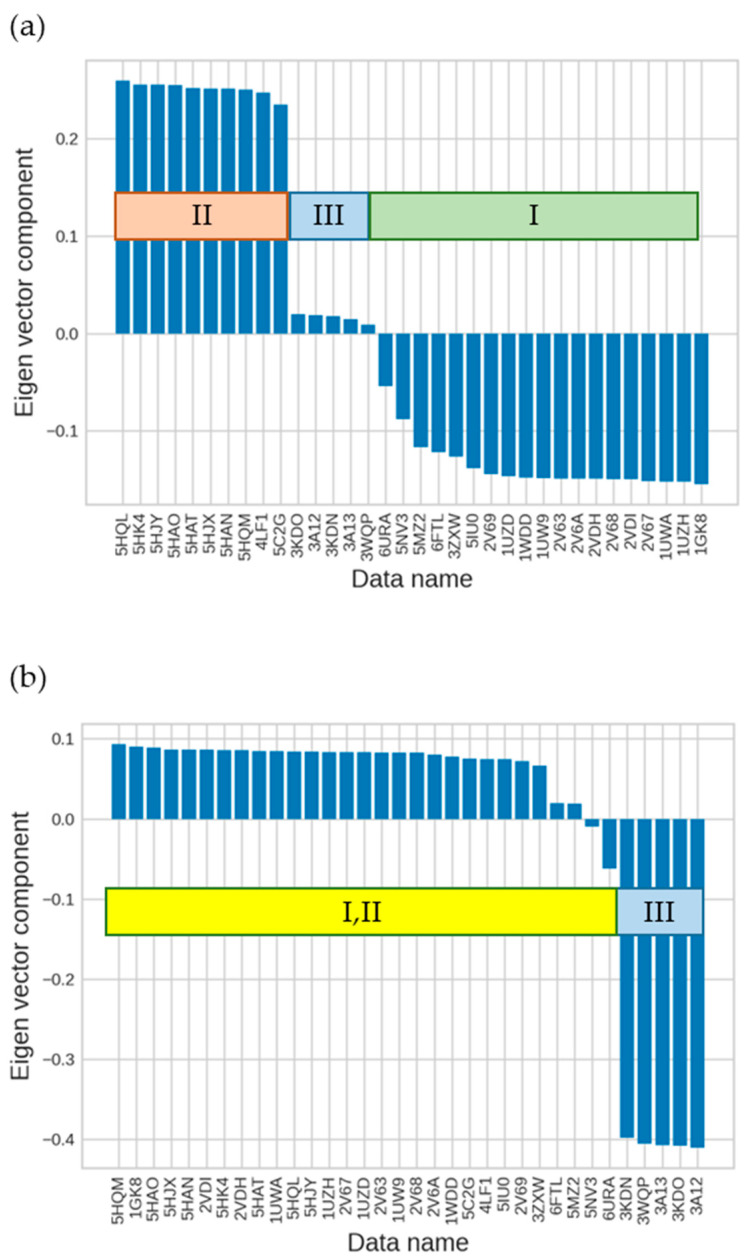
SVD analysis of structures using normalized IFIE data (left-singular vectors). (**a**) The first and (**b**) second left-singular vectors for each structure.

**Figure 13:**
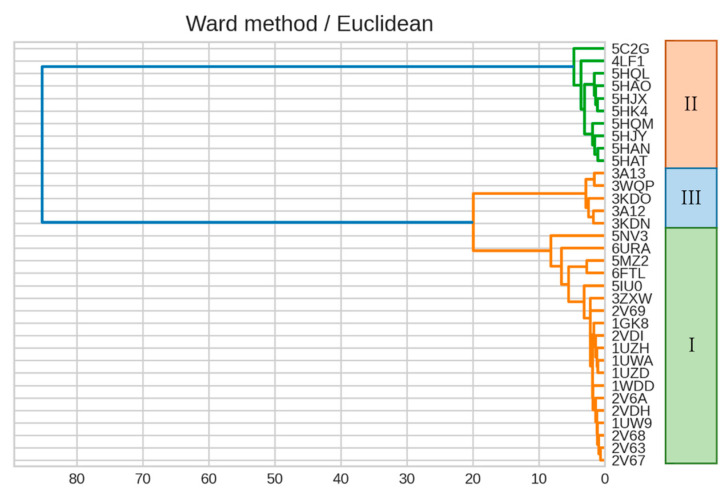
Dendrogram showing the clustering result using Ward’s method. Color coding is set at 60% of the maximum Euclidean distance. The dendrogram was generated using top 120 sites for the second left-singular vector using normalized IFIE data.

The results of SVD showed that the second and third singular components without normalization corresponded to the first and second singular components with normalization, respectively, and that they expressed the difference in forms as well as the difference between form III and forms I and II (Figure 6 and Figure 12). The sites comprising the corresponding features were different for each site (see Table 1 and Table 4, and details are shown in Figures S1 and S3 in Supplementary Materials). Additionally, to determine the number of sites required to reproduce the sequence-based form classification, we increased the number of sites with larger absolute values that made up the first singular vector to 20, 40, and 60 and then reviewed the respective dendrograms. We found that the tree shape was unstable unless ~120 sites were used and that it was impossible to express the classification of forms by sequences (Figure 13). Therefore, we considered that a small number of sites would be insufficient to express the differences in isoforms. Because this result did not differ significantly from that obtained without normalization, we considered no particular merit in using IFIE normalization as a preprocessing step in the present analysis.

## 3. Materials and Methods

### 3.1. FMO Method and IFIE

The FMO method [13,14] is a computational method that efficiently performs ab initio quantum-mechanical calculations for large biomolecules. This method divides large biomolecules, such as proteins, into relatively small units called fragments (usually identified as amino acid residues) and then calculates the energy of the whole molecule and the electron density quantum-chemically by MO calculations of fragments alone (monomers) and fragment pairs (dimers) (sometimes, trimers and tetramers are also considered). The total electron energy of the whole molecular system can be approximated (in the FMO2 approximation) [13,14]:

(2) $$E=\underset{I>J}{\sum }E_{IJ}-\left(N_{f}-2\right)\underset{I}{\sum }E_{I}$$

where ***N*_f_** is the number of fragments, and $E_{I}$ and $E_{IJ}$ are the total electron energies of a fragment (amino acid residue or ligand molecule) and its pair, respectively. If **Δ*P*** is the difference matrix of the electron densities between the monomers and dimer, Equation (2) can be transformed into the following equation:

(3) $$E=\underset{I>J}{\sum }\left({E^{\prime }}_{IJ}-{E^{\prime }}_{I}-{E^{\prime }}_{J}\right)+\underset{I>J}{\sum }Tr\left(\Delta P^{IJ}V^{IJ}\right)+\underset{I}{\sum }{E^{\prime }}_{I}$$

where ${E^{\prime }}_{I}=E_{I}-V_{I}$ , ${E^{\prime }}_{IJ}=E_{IJ}-V_{IJ}$, $V_{I}=Tr\left(P^{I}V^{I}\right),$ and $V_{IJ}=Tr\left(P^{IJ}V^{IJ}\right)$; $V_{I}$ and $V_{IJ}$ are the electrostatic potentials that fragment I and fragment pair IJ receive from surrounding fragments, respectively, and *Tr* refers to the trace. Because this formula contains only the electrostatic potential for the dimer, different approximate electrostatic potentials can be used for the monomers and dimers, and

(4) $$\Delta E_{IJ}=\left({E^{\prime }}_{IJ}-{E^{\prime }}_{I}-{E^{\prime }}_{J}\right)+Tr\left(\Delta P^{IJ}V^{IJ}\right)$$

can be interpreted as the effective interaction energy between fragment pair IJ. This $\Delta E_{IJ}$ is referred to as the IFIE [14,15,17,18,19] and represents the ligand–residue interaction when I and J are assigned for a ligand and a residue, respectively. Furthermore, IFIE-sum is defined as the sum of the IFIEs between a ligand and amino acid residues and can approximate the binding energy between the ligand and protein.

### 3.2. Structure Preparation

**Figure 14:**
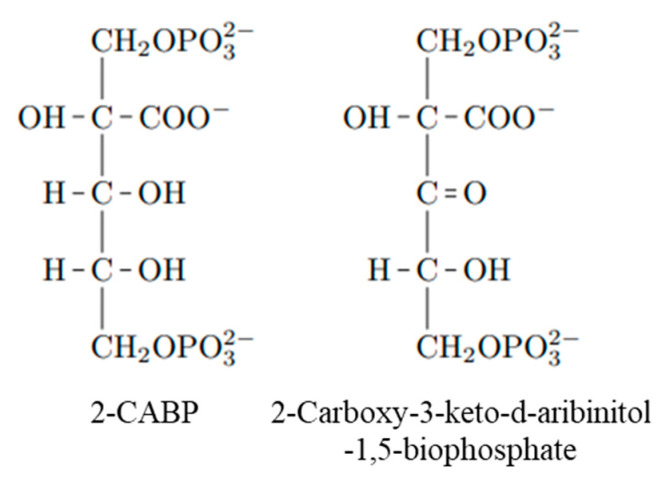
Reaction intermediate analog 2CABP and reaction intermediate 2-carboxy-3-keto-aribinitol-1,5-biophosphate.

In this study, we used 35 three-dimensional structures of RuBisCO in complex with the ligand 2CABP (see Table S1 in Supplementary Materials) from the Protein Data Bank (PDB [20]). Ligand 2CABP is a transition-state analog of the RuBP substrate (Figure 14). The charge of the ligand was set to −5, with two phosphates and a carboxyl group charged to −2 and −1, respectively. Although the entire ligand was heavily negatively charged, we did not observe any problems concerning electrostatic instability because of the positively charged residues and cations around it. For fragmentation around the ligand, we considered a fragmentation method that would allow the calculations to be completed relevantly and the atomic charge of Mg to be close to +2 by natural bond orbital analysis. As a result, the side chains of Asp194 and Glu195, Mg^2+^, and the carboxyl group of 2CABP were gathered into one fragment (see Figure S4 in Supplementary Materials).

The structures were prepared using the molecular operating environment (MOE; v2020.09; Chemical Computing Group Inc., Montreal, QC, Canada [21]) according to the following procedure. First, we removed all water molecules from the crystal structure and all atoms except for the atoms that make up one L_2_ dimer, the 2CABP, and the Mg^2+^ present in its active sites and SSU in Form I. The missing residues and atoms were complemented by the “Structure Preparation” function (built-in functions in MOE), and hydrogen atoms were added using the “Protonate3D” function at pH 7.0. The residues at the N- and C-termini were treated as electrically neutral with NH_2_ and COOH, respectively. Subsequently, only the positions of the complementary atoms (which were missing in the PDB data) and hydrogen atoms were energetically optimized using the Amber10:EHT force field.

The FMO calculation software ABINIT-MP [14] was used to perform FMO calculations with the prepared structures, where the MP2/6-31G* level was employed to treat the electron-correlation effects. The computational time required for each RuBisCO complex (L_2_ dimer) was 8–11 h on 32 nodes of a Fugaku supercomputer. The analogous FMO analysis for the L_8_S_8_ complex was difficult to perform on the present computational platform. The FMO calculation results were registered in FMO database (FMODB [22,23,24]), and their entry IDs (FMODB IDs) are listed in Table S1.

### 3.3. Singular Value Decomposition (SVD)

SVD is a matrix-decomposition method [15]. An m × n matrix, *A*, can be decomposed into the product of three matrices, as shown in Equation (5):

(5) $$A=U\Sigma V^{T}$$

where U is an m × m orthogonal matrix, $V^{T}$ is an n × n orthogonal matrix, and Σ is an m × n diagonal matrix. $\sigma_{ij}$ is an element of Σ, $\sigma_{ij}=0$ if i≠j, and $\sigma_{ij}=\sigma_{i}\geq 0$ if i=j for 1≤i≤n, where $\sigma_{1}\geq \sigma_{2}\geq \sigma_{3}\geq \cdots$. $\sigma_{i}$ is called the singular value of A. The column vector of U is called the left-singular vector, and the row vector of $V^{T}$ is called the right-singular vector.

In this study, a 1115 × 35 IFIE matrix was constructed by arranging 35 PDB-based datasets for the IFIEs of 1115 residues (for L_2_ dimer) interacting with the ligand and used as the column vector of *A*. Because the amino acid sequence number was assigned as the row, and the PDB identifier was assigned as the column in the IFIE matrix, the column vector of U was the orthonormal basis for amino acid residues, and the row vector of $V^{T}$ was the orthonormal basis for the PDB data, whereas each singular vector has an independent meaning.

### 3.4. Ward’s Method

A dendrogram based on the Ward’s method can be constructed by repeating the following procedure until n clusters of size 1 are included into one cluster. Any two clusters $P_{i}$ and $P_{j}$ are combined into a cluster $Q_{ij}$. Then, we define the distance between $P_{i}$ and $P_{j}$ as

(6) $$d_{ij}=L\left(P_{i}\cup P_{j}\right)-L\left(P_{i}\right)-L\left(P_{j}\right)$$

where $L\left(P_{i}\cup P_{j}\right)$ is the sum of the squares of the distances between the center of gravity of $Q_{ij}$ and each sample, and $L\left(P_{i}\right)$ is the sum of the squares of the distances between the center of gravity and each sample in the $P_{i}$ cluster. Then, $d_{ij}$ is calculated for all pairs of clusters, and the cluster of i and j with the smallest $d_{ij}$ is one cluster. This analysis involved 35 PDB-based datasets of IFIEs with 1115 residues interacting with ligands.

### 3.5. Phylogenetic Analysis

Multiple alignments of the sequences to construct the phylogenetic tree were performed using molecular evolutionary genetics analysis (MEGA) X [25]. The analysis involved 35 amino acid sequences corresponding to the data used in the FMO calculations. MUSCLE (multiple sequence comparison by log-expectation) was used as the alignment algorithm [26]. A phylogenetic tree was constructed using maximum likelihood, where the Jones–Taylor–Thornton model [27] was used as the substitution model.

## 4. Conclusions

In summary, we performed FMO calculations on RuBisCO to investigate whether the IFIE matrix is related to the phylogenetic relationships in the sequences or whether there is any information that cannot be obtained only from sequence analysis. Extraction of the features of the IFIE matrix using SVD revealed that the second and third singular vectors represented the difference in forms and the difference between form III and forms I and II, respectively. Moreover, these results did not change significantly after normalizing the IFIE data during preprocessing, suggesting that the differences in sequences were strongly related to those in the interactions with 2CABP. Additionally, examination of the positions of residues with large absolute values of the second singular vector showed the significant roles of residues far away from the 2CABP ligand, indicating that the phylogenetic relationships of residues around the ligand alone differed from those based on whole sequences. This suggested that both the residues proximal to the ligand and those far from the ligand were important for interspecies comparison, which could not be obtained from sequence information alone. Our results thus suggest that substrate-residue interactions can be an essential feature to understand the enzymatic evolution of RuBisCO and may provide a novel insight complementary to that obtained in a recent computational approach [28]. Methodologically, the present scheme can be used to find closely related enzymes with different substrates, which is quite difficult using classical alignment methods.

In this study, we used the L_2_ dimer of RuBisCO for the interspecies comparison mainly due to the limitation of computational cost. The obtained results suggested that even the analysis based on the L_2_ dimer can differentiate the type of isoforms fairly well. Furthermore, by considering the number of oligomerizations, which vary by species, and the effects of water molecules, we may be able to include exceptional 5MAC and other species that could not be analyzed in this study. The FMO analysis for L_8_S_8_ complex of (form I) RuBisCO would be desirable for future research. In addition to the phylogenetic relationships, analysis of the IFIE matrix may provide new insights into the evolution of RuBisCO in relation to the reaction rates and substrate specificity, whose details also remain to be elucidated.

## Figures and Tables

**Table 1:** Top eight sites with the largest absolute values of eigenvector elements in the first, second, and third left-singular vectors of SVD.

First	204	206	230	302	329	370	631	1115
Second	332	451	533	536	582	627	708	710
Third	183	244	252	383	514	529	532	708

**Table 2:** Top eight sites with the largest average absolute values of IFIE.

Rank	1	2	3	4	5	6	7	8
Residue site	329	370	204	631	230	206	1115	332

**Table 3:** Top eight sites of the second left-singular value in Table 1 and corresponding residues for each form. Where there are multiple residues, the first amino acid residue is the most common in that form.

	332	451	533	536	582	627	708	710
Form I	H, N	H, Q	I, N	E, D, N	K, E, Q	A	A, P	R, K, S, A, G
Form II	H	R	D	K	D	H	D	E, A
Form III	H	N	V	V	D	A	R	K

**Table 4:** Top eight sites with the largest absolute values of eigenvector elements in the first and second left-singular vectors of SVD.

First	95	148	270	340	344	398	699	900
Second	170	183	252	264	280	529	739	955

## Data Availability

All the data are available, and the calculation results are registered in FMO database (FMODB [22]).

## Supplementary Materials

The following supporting information can be downloaded at: https://www.mdpi.com/article/10.3390/ijms231911347/s1.
