# Supplementary material for: Interspecies Comparison of Interaction Energies between Photosynthetic Protein RuBisCO and 2CABP Ligand
Source: Int J Mol Sci. 2022 Sep 26;23(19):11347. doi: 10.3390/ijms231911347 (PMC9570433; doi:10.3390/ijms231911347)
Supplement: Supplementary file 1 [file ijms-23-11347-s001.zip › ijms-1901948-supplementary.pdf]

International Journal of
Molecular Sciences

MDPI

Supplementary Materials

Interspecies Comparison of Interaction Energies between Photosynthetic Protein RuBisCO and 2CABP Ligand

Masayasu Fujii and Shigenori Tanaka*

Department of Computational Science, Graduate School of System Informatics, Kobe University, 1-1 Rokkodai, Nada-ku, Kobe 657-8501, Japan
* Correspondence: tanaka2@kobe-u.ac.jp

Table S1. PDB-IDs and FMODB-IDs used for phylogenetic analyses and FMO calculations.

PDBID	[a]FMODB ID	Organism	Form	Mutations
1GK8	VRQN1	*Chlamydomonas reinhardtii*	I	None
1UW9	YYV12	*Chlamydomonas reinhardtii*	I	L290F,A222T
1UWA	53J5Z	*Chlamydomonas reinhardtii*	I	L290F
1UZD	43JYN	*Chlamydomonas reinhardtii*	I	None
1UZH	K3GV3	*Chlamydomonas reinhardtii*	I	None
1WDD	Q1VLY	*Oryza sativa Japonica Group*	I	None
2V63	R58L8	*Chlamydomonas reinhardtii*	I	V331A,L46P
2V67	ZYVZN	*Chlamydomonas reinhardtii*	I	T342I
2V68	66Y4Z	*Chlamydomonas reinhardtii*	I	V331A,T342I
2V69	22NMR	*Chlamydomonas reinhardtii*	I	D473E
2V6A	7G6KK	*Chlamydomonas reinhardtii*	I	V331A,G344S
2VDH	M3NLZ	*Chlamydomonas reinhardtii*	I	C172S
2VDI	9G8R2	*Chlamydomonas reinhardtii*	I	C192S
3A12	LJNV9	*Thermococcus kodakarensis*	III	None
3A13	3QJML	*Thermococcus kodakarensis*	III	G326E,K327R,W328D,D329I,V330T
3KDN	J3QV9	*Thermococcus kodakarensis*	III	G326E,K327R,W328D,D329I,V330T
3KDO	N1NLQ	*Thermococcus kodakarensis*	III	G326E,K327R,W328D,D329I,V330T, I331L,Q332G,N333F,A334V,R335D,I336L
3WQP	829QY	*Thermococcus Kodakarensis*	III	T289D
3ZXW	GN5V1	*Thermosynechococcus elonagatus*	I	None
4LF1	14N9Z	*Rhodopseudomonas palustris*	II	None
5C2G	VRQY1	*Gallionella*	II	None
5HAN	YYVR2	*Rhodopseudomonas palustris*	II	S59F
5HAO	53JGZ	*Rhodopseudomonas palustris*	II	M331A

5HAT	43JGN	*Rhodopseudomonas palustris*	II	S59F/M331A
5HJX	K3GQ3	*Rhodopseudomonas palustris*	II	A47V
5HJY	Q1V5Y	*Rhodopseudomonas palustris*	II	I165T
5HK4	R5848	*Rhodopseudomonas palustris*	II	A47V,M331A
5HQL	ZYV9N	*Rhodopseudomonas palustris*	II	A47V,M331A
5HQM	66Y5Z	*Rhodopseudomonas palustris*	II	None
5IU0	22NGR	*Arabidopsis thaliana*	I	None
5MAC	7G64K	*Methanococcoides burtonii*	II	None
5MZ2	M3NZZ	*Thalassiosira antartica var. borealis*	I	None
5NV3	9G8Q2	*Rhodobacter sphaeroides*	I	None
6FTL	LJNR9	*Sleletonema marinoi*	I	None
6URA	3QJGL	*Candidatus Promineofilum breve*	I	None

[a]The FMO calculation results are registered in FMO database (https://drugdesign.riken.jp/FMODB/) and their entry IDs (FMODB IDs) are listed.

(a)

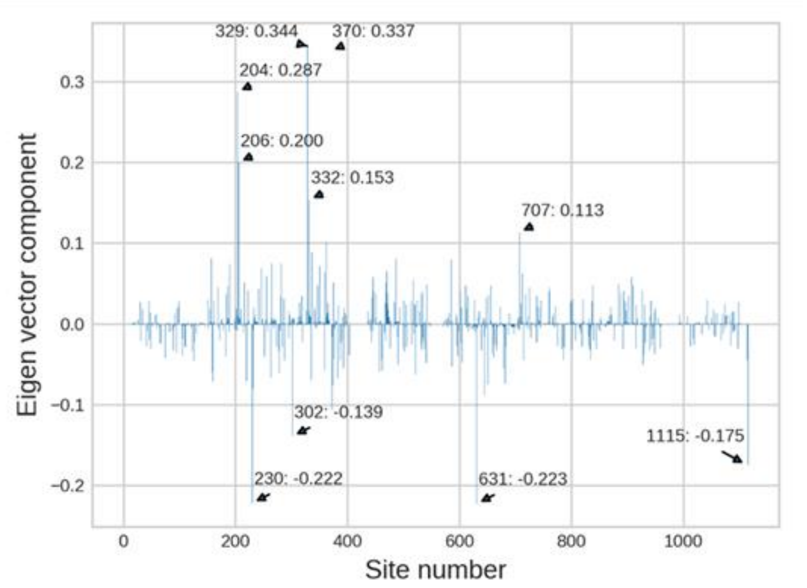

(b)

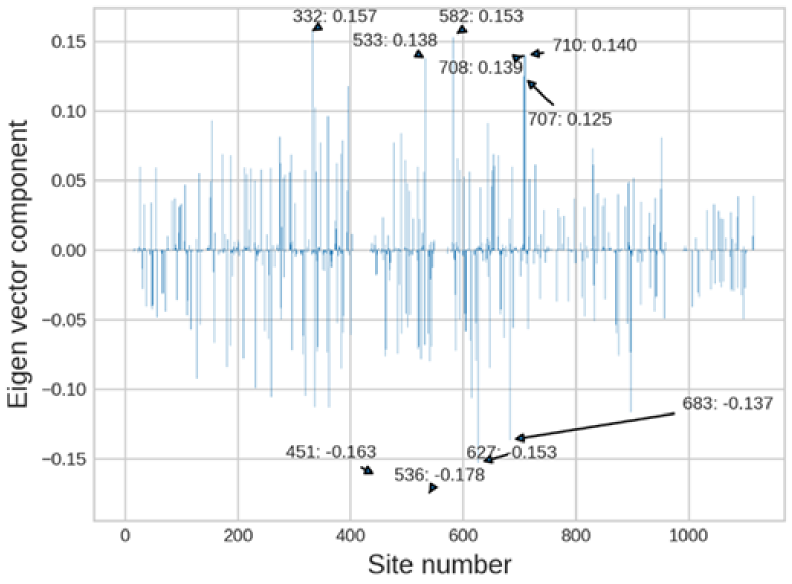

(c)

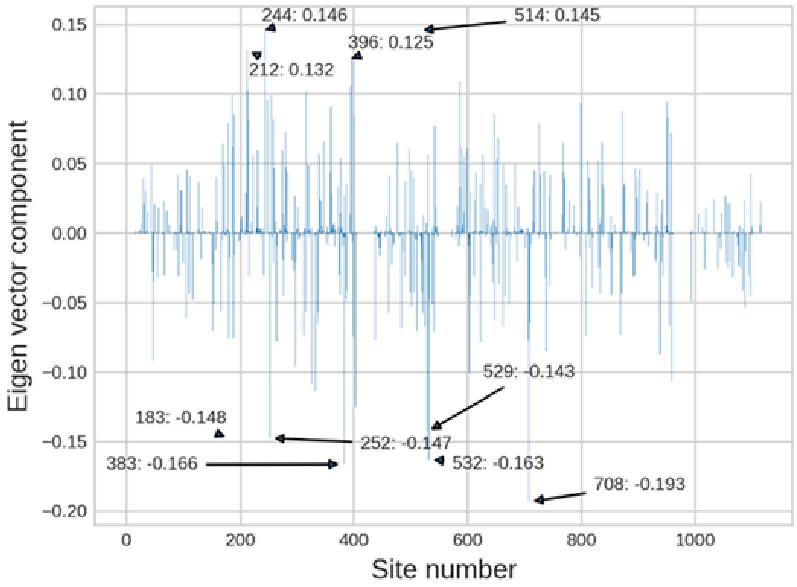

Figure S1. Results of SVD analysis for the left-singular vectors of IFIE. (**a**) The first, (**b**) second, and (**c**) third left-singular vectors for each residue site.

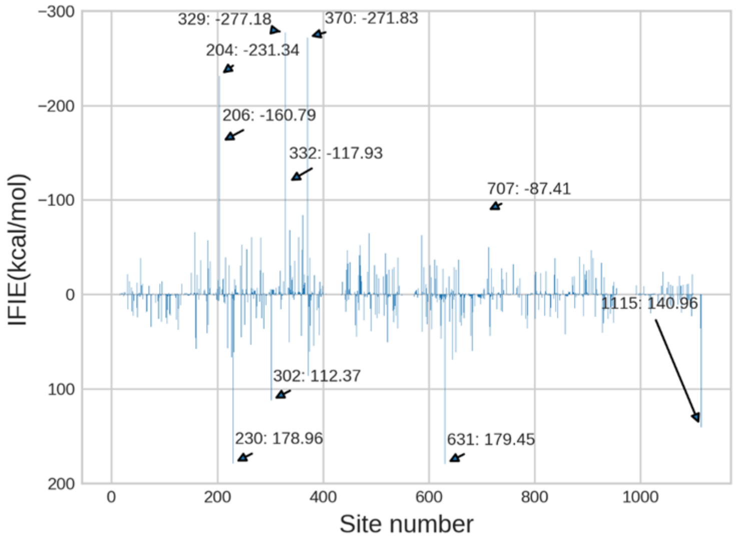

Figure S2. IFIEs for each residue site averaged over 34 complexed structures. Positive and negative values of IFIEs indicate the repulsive and attractive interactions, respectively.

(a)

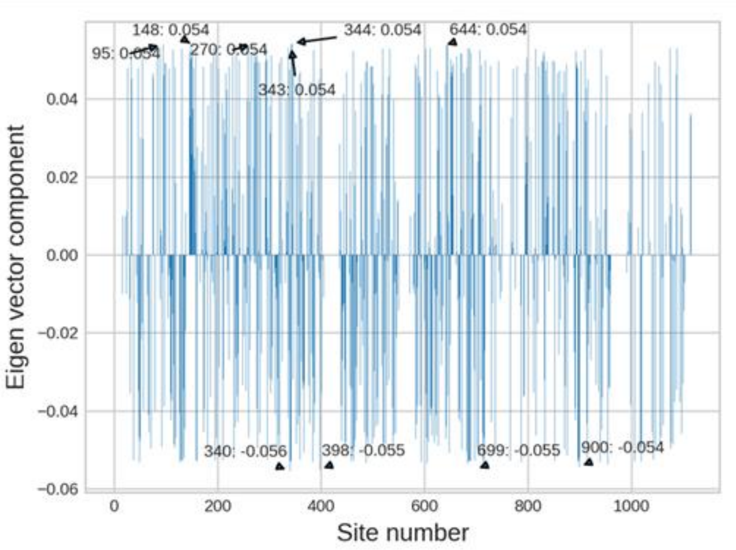

(b)

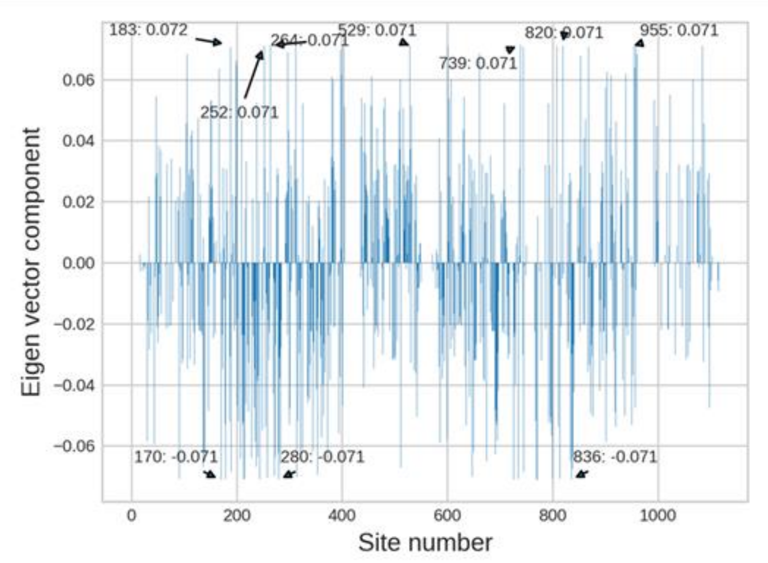

Figure S3. SVD analysis for residue sites using normalized IFIE data (left-singular vectors) (**a**) The first and (**b**) second left-singular vectors for residue site number.

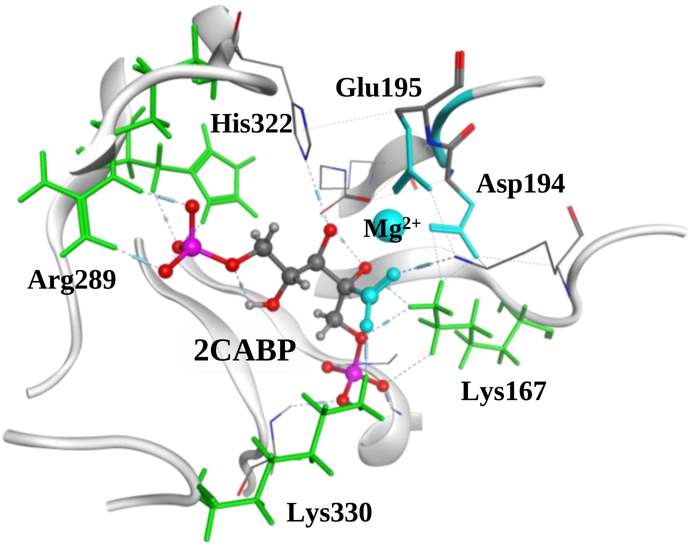

Figure S4. The active site surrounding the 2CABP ligand (PDB ID: 4LF1, FMODB ID: 14N9Z), which is represented by ball and stick model. Cyan sticks refer to the side chains of Asp194 and Glu195 and the carboxyl group of 2CABP, all of which belong to the same fragment as Mg^{2+} represented by a cyan sphere. Green sticks refer to positively-charged residues in the vicinity of the ligand.
